# H_2_O_2_ Solution Steaming Combined Method to Cellulose Skeleton for Transparent Wood Infiltrated with Cellulose Acetate

**DOI:** 10.3390/polym15071733

**Published:** 2023-03-31

**Authors:** Jun Zhang, Yongling Ying, Xiaoyang Yi, Wenbo Han, Lu Yin, Yongjun Zheng, Rongbo Zheng

**Affiliations:** 1 School of Materials and Chemical Engineering, Southwest Forestry University, Kunming 650224, China; 2 School of Marine Science and Technology, Shanwei Institute of Technology, Shanwei 516600, China

**Keywords:** H_2_O_2_ solution steaming delignification, transparent wood, cellulose acetate

## Abstract

Hydrogen peroxide (H_2_O_2_) steaming, a green and highly efficient delignification method, has been demonstrated to provide a wood skeleton with a very low content of residual lignin in the manufacturing of transparent wood. It usually requires a long reaction time and a large amount of H_2_O_2_ because the piece of wood is treated using steaming equipment. Herein, a H_2_O_2_ solution steaming method was developed for the highly efficient removal of lignin from wood. Specifically, several wood samples were simultaneously immersed in a hot H_2_O_2_ solution to obtain delignified wood with a relatively high content of residual lignin, which provided a high strength and preserved the cellulose skeleton. Subsequently, the delignified wood with a relatively high content of residual lignin was further treated with H_2_O_2_ steam to obtain a very low lignin delignified wood. Compared with the previous H_2_O_2_ steaming method, the reaction time and used H_2_O_2_ volume of the H_2_O_2_ solution steaming method was reduced by 37.3% and 52.7%, respectively. All-biomass transparent wood could be obtained by infiltrating the delignified wood with cellulose acetate, which showed both a high transmittance of 83.0% and a low thermal conductivity of 0.30 Wm^−1^K^−1^.

## 1. Introduction

Transparent wood has potential applications in energy-efficient buildings and electronic devices, owing to its high optical transmittance, low thermal conductivity, and high safety [1,2]. Various delignification methods have been developed to remove lignin from wood, including sodium chlorite (NaClO_2_) [1], sodium hypochlorite (NaClO) [3], sodium hydroxide/sodium thiosulfate (NaOH/NaS_2_O_3_) [2], and hydrogen peroxide (H_2_O_2_) steaming [4], all of which provide well-preserved cellulose skeletons for transparent wood through infiltration with epoxy resin, PMMA, PVA, PLA, and PVP [5,6,7,8,9]. Previous studies have demonstrated that H_2_O_2_ steaming, a green and highly efficient delignification method, provides a wood skeleton with a very low content of residual lignin, which could be used further as a skeleton for transparent wood [4,10], catalyst supports [11], solar evaporation generators [12], and bioplastics [13,14]. This method, however, has long reaction times and consumes a large amount of H_2_O_2_, because delignification is only achieved by H_2_O_2_ steaming, which requires a large volume of boiling H_2_O_2_ solution to generate steam. Although it is highly efficient for delignification, common methods usually require steaming equipment to treat a piece of wood, which consumes more H_2_O_2_ and requires a longer reaction time. Therefore, it remains challenging to develop a high-efficiency H_2_O_2_ delignification method for a cellulose skeleton for the manufacturing of transparent wood.

It is well known that the mechanical strength of delignified wood decreases as the content of residual lignin is reduced. That is, delignified wood with high lignin content can remain well preserved in a hot H_2_O_2_ solution, whereas delignified wood with a very low lignin content can remain well preserved in steaming equipment [4]. In this work, a H_2_O_2_ solution steaming method was developed to achieve the highly efficient removal of lignin from transparent wood. Several wood samples could be simultaneously immersed in hot H_2_O_2_ solution to obtain delignified wood with a relatively high content of residual lignin, which provides a high strength to preserve the cellulose skeleton. Subsequently, the delignified wood with a high lignin content was treated with H_2_O_2_ vapor to obtain ultra-low lignin delignified wood. Compared with the previous H_2_O_2_ steaming method, the reaction time and H_2_O_2_ volume of the H_2_O_2_ solution steaming method was reduced by 37.3% and 52.7%, respectively. In addition, in this work, cellulose acetate, which is a cellulose derivative, was used for the first time to fill the cellulose backbone of wood, and cellulose-based transparent wood was prepared by a top-down method. All transparent wood could be obtained by infiltrating the delignified wood with cellulose acetate, which showed both a high haze (91.0%) and a high light transmittance (83.0%), leading to potential applications in antiglare windows. The cellulose acetate transparent wood (CATW) had a good tensile strength (56.2 MPa) and modulus (1.87 GPa) suitable for daily applications. The CATW possessed a low thermal conductivity (0.30 Wm^−1^K^−1^), which demonstrate it as an excellent insulating material and a candidate for energy-efficient buildings.

## 2. Experimental

### 2.1. Materials and Chemicals

In the present work, cellulose acetate transparent wood (CATW) with a density of 0.98 ± 0.05 g cm^−3^ was made from balsa wood. The chemical used to remove lignin from the wood was H_2_O_2_ (30% solution, Sinopharm Chemical Reagent Co., Ltd., Shanghai, China). Cellulose acetate (bound acetic acid, 54.5–56.0%, viscosity, 300–500 mPa·s, Sinopharm), ethyl acetate, and ethanol were purchased from Macklin (Shanghai, China).

### 2.2. Delignification of the Wood

**Scheme 1:**
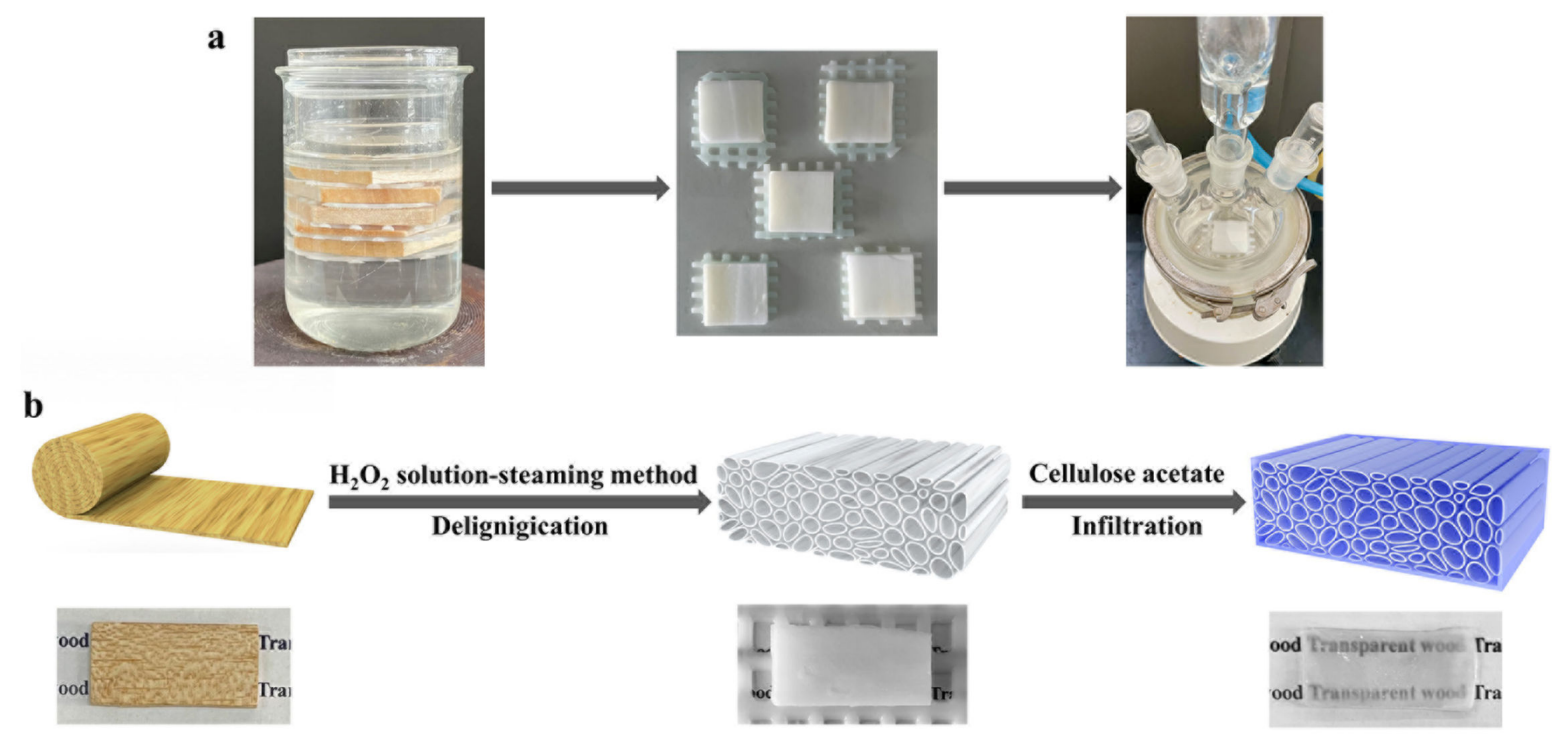
(**a**) Process diagram for the preparation of H_2_O_2_ solution steaming of bleached wood. (**b**) Preparation process of cellulose acetate transparent wood.

First, five sheets of 40 × 20 × 1 mm^3^ balsa wood were immersed in a beaker containing 30% H_2_O_2_ solution at ~95 °C for about 7.5 h. Next, a piece of the balsa wood sample was places into the specific device (Scheme 1a) containing the H_2_O_2_ steam generated from a 30% H_2_O_2_ solution at a temperature of 95 °C for about 2.5 h. The H_2_O_2_ steam penetrated the wood cell walls and continued to remove lignin until the yellow color of the wood was completely removed. Finally, the delignified wood was sequentially rinsed three times with deionized water, ethanol, and ethyl acetate to remove residual chemicals and impurities generated during the delignification and to remove water from the wood to obtain delignified wood sheets.

### 2.3. Synthesis of Cellulose Acetate Transparent Wood

First, a cellulose acetate solution was prepared by dissolving cellulose acetate powder (8–10 wt%) in a mixture of ethanol:ethyl acetate = 1:3 (*v*/*v*) by stirring. The bleached wood sheets were placed in a beaker and immersed in the cellulose acetate solution at a depth of approximately 15 times more than the thickness of the wood. Next, the beaker was placed in a vacuum desiccator at room temperature. The solution was degassed using solvent evaporation to allow cellulose acetate to penetrate into the sheets, which were maintained at room temperature under vacuum (0.05 MPa) until the ethanol and ethyl acetate in the solution were completely evaporated. Then, the obtained cellulose acetate transparent wood was removed from the beaker. Finally, to obtain cellulose acetate transparent wood, the obtained samples were hot-pressed at 55–60 °C and 15 MPa for 3 h. A summary of the cellulose acetate transparent wood preparation process is shown in Scheme 1b.

### 2.4. Measurements and Characterizations

The morphology of the samples was analyzed using scanning electron microscopy (SEM; TESCAN VEGA3, Brno, Czech Republic). The cellulose, hemicellulose, and lignin content measurements followed standard methods (Pulp and Paper Industry Technical Association Standard Method T 222-om-83). The mechanical properties of the samples were measured using a universal testing machine (ProLine Z020TN, Zwick, Ulm, Germany). The transmittance spectra and haze were measured with an ultraviolet-visible spectrophotometer (Hitachi, U-101 4100, Hitachi, Tokyo, Japan). A tensor 27 FTIR spectrometer (Bruker Karlsruhe, Germany) was used to acquire Fourier transform infrared (FTIR) spectra in the range of 400–4000 cm^−1^. The scanning frequency was 10 kHz and the scanning time was 60 s. The measurement of thermal properties followed two methods: (1) qualitative measurements of insulation properties under conductive heat sources and (2) thermal conductivity measurements. The upper-surface temperature of the samples was measured with an infrared thermographer (UNI-T, UTi120s, UNI-T, Dongguan, China) when the upper-surface temperature had reached a steady state. The thermal conductivity of the samples was measured using a thermal constant analyzer (Hot Disk, TPS2500S, Hot Disk, Gothenburg, Sweden).

## 3. Results and Discussion

Balsa wood is the least dense tree species due to its fast growth. Balsa wood samples with a thickness of about 1 mm were cut along the axial direction of tree growth. The original wood was opaque because of the presence of light-absorbing lignin inside the wood (which intensely absorbs light in the visible spectral region from 380 to 780 nm, causing the fibers to exhibit a specific color) and because of the presence of air in the pores of the fibers inside the wood [6]. Air has a refractive index of 1.00, whereas cellulose and hemicellulose have a refractive index of about 1.53, which results in strong light scattering when light hits the fibers, leading to the opacity of the natural fibers [15].

**Figure 1:**
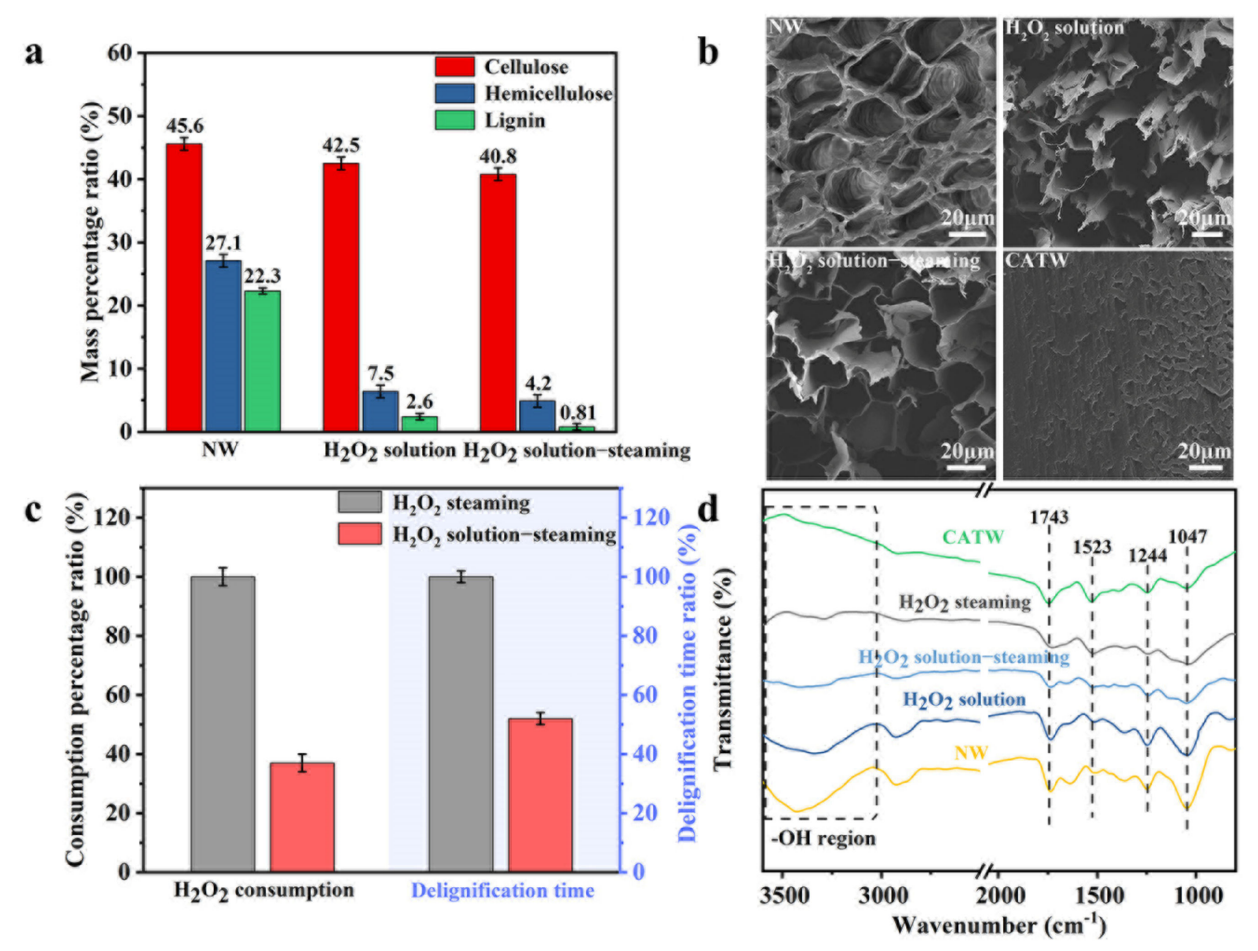
(**a**) Cellulose, hemicellulose, and lignin contents of natural wood (–NW), H_2_O_2_ solution bleached wood, and H_2_O_2_ solution steaming bleached wood. (**b**) SEM images of NW, H_2_O_2_ solution bleached wood, H_2_O_2_ solution steaming bleached wood, and CATW. (**c**) Consumption percentage ratio and delignification time ratio determined for the H_2_O_2_ steaming method and the H_2_O_2_ solution steaming method. (**d**) FTIR spectra of NW, H_2_O_2_ solution bleached wood, H_2_O_2_ solution steaming bleached wood, H_2_O_2_ steaming method bleached wood, and CATW.

In this study, to prepare transparent wood, the H_2_O_2_ solution steaming method was used to remove lignin and hemicellulose from the wood. The H_2_O_2_ solution steaming method was an optimized version of the H_2_O_2_ steaming method used in previous work [4]. In the H_2_O_2_ system, some reactive anions and radicals are easily formed from the H_2_O_2_ molecule, such as hydrogen peroxide (HOO^−^) ions and superoxide (O_2_^−^) and hydroxide (HO^·^) radicals. Among these anions and radicals, HOO^−^ ions are the main group that react with lignin by cleaving the bonds between the aromatic rings and side chains. Additionally, the HOO^−^ ions further break the unsaturated bonds in the side chains of the lignin molecule, such as the carbonyl and olefinic aldehyde groups, which leads to the formation and subsequent decomposition of the ethylene oxide intermediate into small aliphatic molecules. At the same time, aromatic molecules are also attacked by HOO^−^ ions to form epoxide intermediates, which are finally decomposed by oxidation to end products consisting mainly of carbonyl and carboxyl compounds. Similar to HOO^−^ ions, HO^−^ ions and HO^·^ and O_2_^−·^ radicals, in addition, contribute to the oxidation, hydrolysis, and degradation of lignin for the purpose of wood delignification [16]. To demonstrate the merits of this new strategy, the delignification process of 40 × 40 × 5 mm^3^ L-shaped nature balsa wood was analyzed to determine the content of lignin, hemicellulose, and cellulose and the microstructure of the three major elements, as shown in Figure 1. Figure 1a shows that the original natural wood was composed of 45.6% cellulose, 27.1% hemicellulose, and 22.3% lignin. A complete and well-arranged structure of the tubular walls of the wood cross-section was observed by scanning electron microscopy (SEM) (Figure 1b). First, after using a H_2_O_2_ solution to delignify the lignin for about 7.5 h, the lignin content decreased to 2.6% and the sample turned almost white. It was observed in the SEM image that the original pipe wall structure was slightly damaged, and the pipe wall structure was thinner. The cracks on the surface of the sample, which are evident in the photograph, may have been due to the bursting of air bubbles during the whitening of the wood, as shown in Scheme 1a. Next, H_2_O_2_ solution steaming was further used to remove the lignin from the samples. After about 2.5 h, the color turned completely white, and the pipe wall structure of the samples was further damaged and thinned; however, the shape of the pipe wall was retained. The remaining cellulose content of the samples was 40.8%, whereas the hemicellulose and lignin content decreased to 4.2% and 0.81%, respectively. Thus, it was proven that the delignified wood skeleton was still well preserved. Meanwhile, the average H_2_O_2_ consumption and time taken per piece of bleached wood prepared by H_2_O_2_ steaming and H_2_O_2_ solution steaming were calculated and compared. The H_2_O_2_ steaming required about 7.5 h and 375 mL of H_2_O_2_ per piece of bleached wood (x_1_), and the solution steaming in this work required about 3.9 h and 170 mL of H_2_O_2_ per piece of bleached wood (x_2_). The consumption percentage ratio was calculated according to Equation (1).

Consumption percentage ratio (%) = x_2_/x_1_ × 100% (1)

where x_1_ is the consumption of H_2_O_2_ (mL) or time (h) in the H_2_O_2_ steaming method, and x_2_ is the consumption of H_2_O_2_ (mL) or time (h) in the H_2_O_2_ solution steaming method.

Taking the steaming time and chemical usage in the previous work as a baseline (~100%), it was calculated that this method saved about 62.7% and 47.3% in terms of time and H_2_O_2_ consumption, respectively, as shown in Figure 1c.

To obtain transparent wood with good light transmission and haze, the bleached wood was filled with cellulose acetate (CA). CA is a degradable cellulose derivative with optical transparency and is widely used in composite materials [17,18,19]. In addition, CA has good wettability compared to cellulose and matches well with cellulose (n = 1.53) with a good refractive index (n = 1.47) [20]. Enabling the full penetration of cellulose derivatives into the delignified wood structure enables a high transparency. The SEM images of the natural wood, the wood after the H_2_O_2_ solution steaming process, and the cellulose acetate transparent wood (CATW) were compared, as shown in Figure 1b. It was observed that the CATW was fully bonded and the cellulose tube walls were well filled with CA after the hot-press treatment. The prepared CATW (Scheme 1b) is clearly labelled in the presented images.

To further confirm the effects of the bleaching and filling processes, the chemical compositions of natural wood (NW), delignified wood, and CATW were analyzed. Figure 1d shows the FTIR spectra of untreated wood, H_2_O_2_ solution-steaming-treated wood, original H_2_O_2_ steaming-treated wood, and CATW. First, the peak at 1523 cm^−1^ was caused by the benzene ring skeleton in the lignin [21]. The peak at 1743 cm^−1^ belongs to the carboxyl group in hemicellulose (xylan/glucomannan) and the peak at 1244 cm^−1^ belongs to the uronic acid group of hemicellulose or the ester linkage of the carboxylic acid group of lignin and hemicellulose [22]. By comparing the NW, H_2_O_2_ solution, and H_2_O_2_ solution-steaming-treated wood, it was found that the peaks at 1523 cm^−1^, 1743 cm^−1^, and 1244 cm^−1^ all decreased significantly and gradually as the wood was bleached twice by boiling and steam. This result proved that the lignin and hemicellulose inside the wood were continuously removed. The stronger peak at 1047 cm^−1^, however, is the characteristic peak of C-O vibration in cellulose [13,14], and the peak at this position decreased but did not change particularly significantly. This result demonstrated that the method had little effect on cellulose, and it mainly affected the removal of lignin and hemicellulose. The peaks of H_2_O_2_ steamed wood from previous work and the H_2_O_2_ solution-steaming-treated wood in this work were compared and were found to be similar, which proved that the bleaching effect of both methods on wood was similar. According to this analysis and a comparison of natural and delignified wood, all curves were further analyzed and compared. The comparison results showed that the introduction of CA resulted in peaks at 1244 cm^−1^ and 1743 cm^−1^. The peak at 1244 cm^−1^ was due to the stretching vibration of the C-O-C bond in CA, and the peak at 1743 cm^−1^ was due to the contraction vibration of the C=O of the ester group added to CA. Meanwhile, the characteristic peak area of -OH was observed in the region of 3000–3500 cm^−1^. Comparing the original wood, the wood treated according to the two delignification methods, and the CATW, it was found that the decreasing -OH peak was due to the removal of lignin and hemicellulose during the bleaching process. Then, the penetration of CA into the delignified wood scaffold by hydrogen bonding caused the -OH peak to decrease again. This explains why the -OH peak decreased.

**Figure 2:**
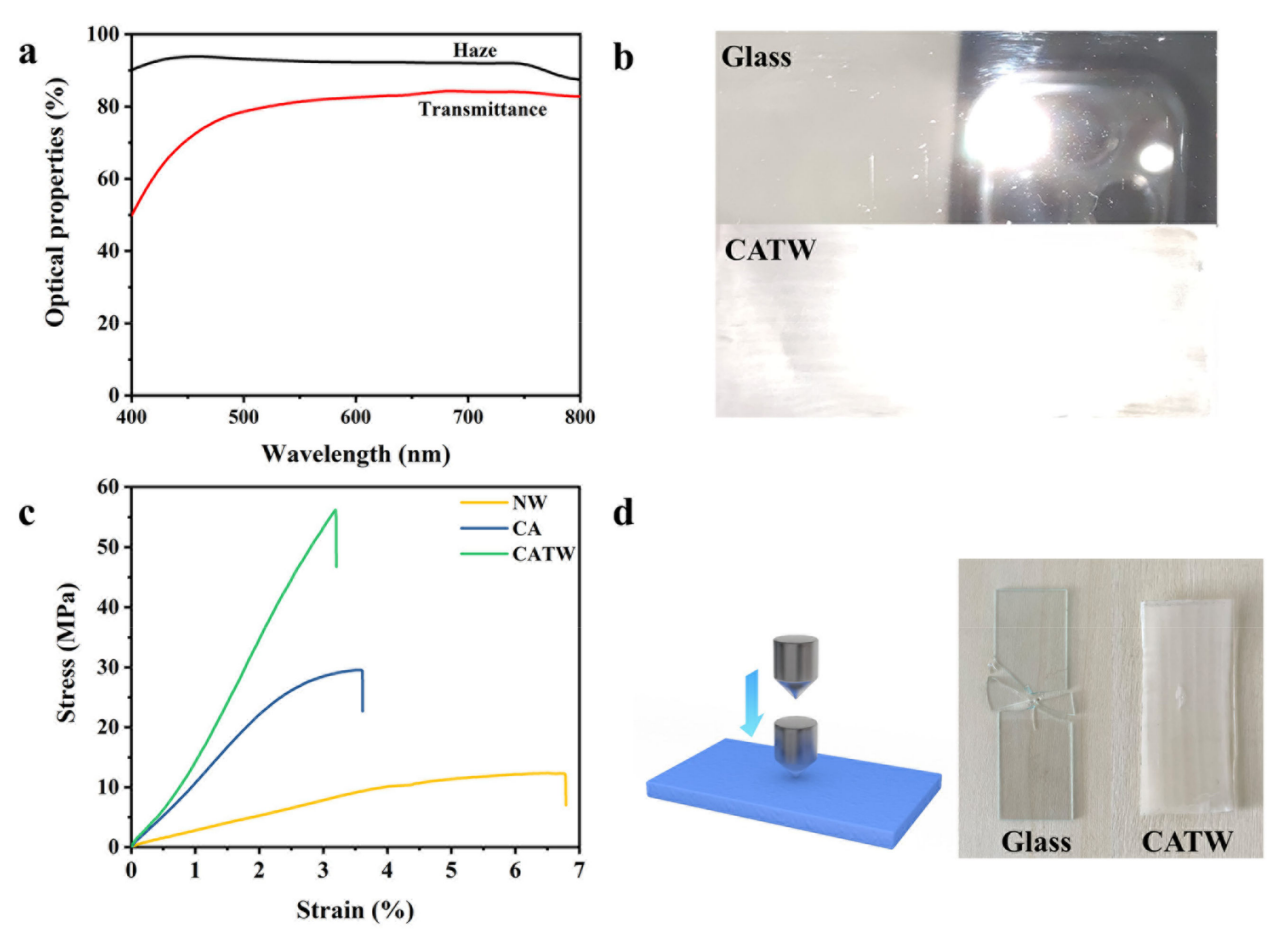
(**a**) Optical transmittance and haze profiles of CATW (1 mm). (**b**) Photograph of the glare effect of glass and CATW. (**c**) Stress–strain curves for NW, CA, and CATW. (**d**) Impact tests of glass and CATW: the glass shatters under a sudden impact (**left** photo) and the CATW shows only a dent on the surface (**right** photo).

The total transmittance and haze of the CATW in the wavelength range of 400–800 nm were measured by an ultraviolet (UV)-visible spectrophotometer (Figure 2a). With a high transmittance of 83.0% at 650 nm, the CATW achieved a haze of 91.0%, which is much higher than that of ultrafast nanoparticles, which have a typical haze value of about 60.0% [5]. Previously reported work has showed that the prepared the delignified wood and the transparent wood prepared by the H_2_O_2_ steaming method and epoxy resin infiltration had a light transmission of 87% at a wavelength of 550 nm and a haze of 90% [4]. According to the literature, a completely biobased transparent wood (TW) was prepared by introducing cellulose nanofiber (CNF) and chitosan (CTS) suspensions into the bleached wood. The prepared TW exhibited an 80% total transmittance and 30–60% haze [7]. The cellulose acetate transparent wood (CATW) prepared in this work has 83% transmittance and 91% haze. In terms of optical properties, the CATW has demonstrated good potential in the field of daylight-covered construction materials because it has similar properties to the transparent wood reported above in the literature. Glare is the appearance of excessive brightness in a localized area of the visual field or changes in excessive brightness. Glare can cause visual discomfort. Sources of glare include the position of the sun in the morning and evening, ice, reflective surfaces on cars, highly polished floors, and windows in nearby buildings. When used for everyday applications, CATW provides effective glare protection because of its high haze and high transmittance properties. To verify the anti-glare effect of CATW, a cell phone light was used as the light source for the test. In comparison to glass, it was found that glare was largely eliminated when light passed through the CATW, and a more uniform brightness was obtained, as shown in Figure 2b. Additionally, the light generated by the light source was more uniform after passing through the CATW. Therefore, this material has demonstrated a high potential in the field of daylight-covered construction materials. For example, when CATW is used as a skylight, it also can maintain a consistent light distribution to the interior throughout the day. In summary, CATW building materials can be effective in saving energy for interior lighting and providing a uniform illumination with enhanced visual comfort and privacy.

In addition to the investigation of its optical characteristics, the mechanical characteristics of CATW were also investigated. To measure the mechanical properties of the samples, each sample was cut into a strip with dimensions of 50 mm × 10 mm × 1 mm for tensile testing. Figure 2c shows the tensile stress–strain curves for NW, CA, and CATW. Compared with NW (Young’s modulus of 3.81 GPa, 12.3 MPa at break) and CA (Young’s modulus of 0.97 GPa, 29.6 MPa at break), the mechanical properties of CATW were improved, reaching 56.2 MPa and 1.87 GPa. This increase in strength is attributed to the reduction in voids within the cellulose after hot pressing and the strong interaction between the cellulose fiber structure and CA through hydrogen bonding. A previously reported work dispersed Cs_x_WO_3_ nanoparticles in prepolymerized methyl methacrylate (PMMA) and used them to fill in the nanopores of delignified wood to prepare transparent wood. The Cs_x_WO_3_/transparent wood had a fracture strength of up to 59.8 MPa and a Young’s modulus of up to 2.72 GPa [8]. It has been reported in the literature that transparent fiber wood can be prepared by a bottom-up method using MMA impregnated fibers and the mold sealing method. The transparent fiber wood exhibited a rupture strength and modulus of 46.8 MPa and 2.2 GPa, respecitvely [15]. The cellulose acetate transparent wood (CATW) prepared in this work had a tensile strength of 56.2 MPa and a modulus of 1.87 GPa. In terms of its mechanical properties, the CATW has similar properties to the transparent wood reported in the literature and described above. In addition to the properties just discussed, glass is used as a building material in residential and commercial buildings. When it experiences a sudden impact, such as that of an external substance, the glass may break and eject shards in all directions. As a result of its high fracture strength of 420.5 MPa and modulus of 62.55 GPa, glass is an ideal brittle material [8]. Glass breakage requires immediate maintenance and attention, because broken glass poses serious safety concerns. CATW can withstand a higher impact than glass. Figure 2d shows the morphology of the glass and CATW after breaking, caused by the sudden impact of a falling sharp object. The glass immediately shattered into sharp fragments, whereas the transparent wood remained intact. Thus, it was demonstrated that transparent wood is a safe, nonhazardous, shatter-resistant, and transparent building material. The high fracture strength and modulus of transparent wood are ideal for heat-shielding window applications.

**Figure 3:**
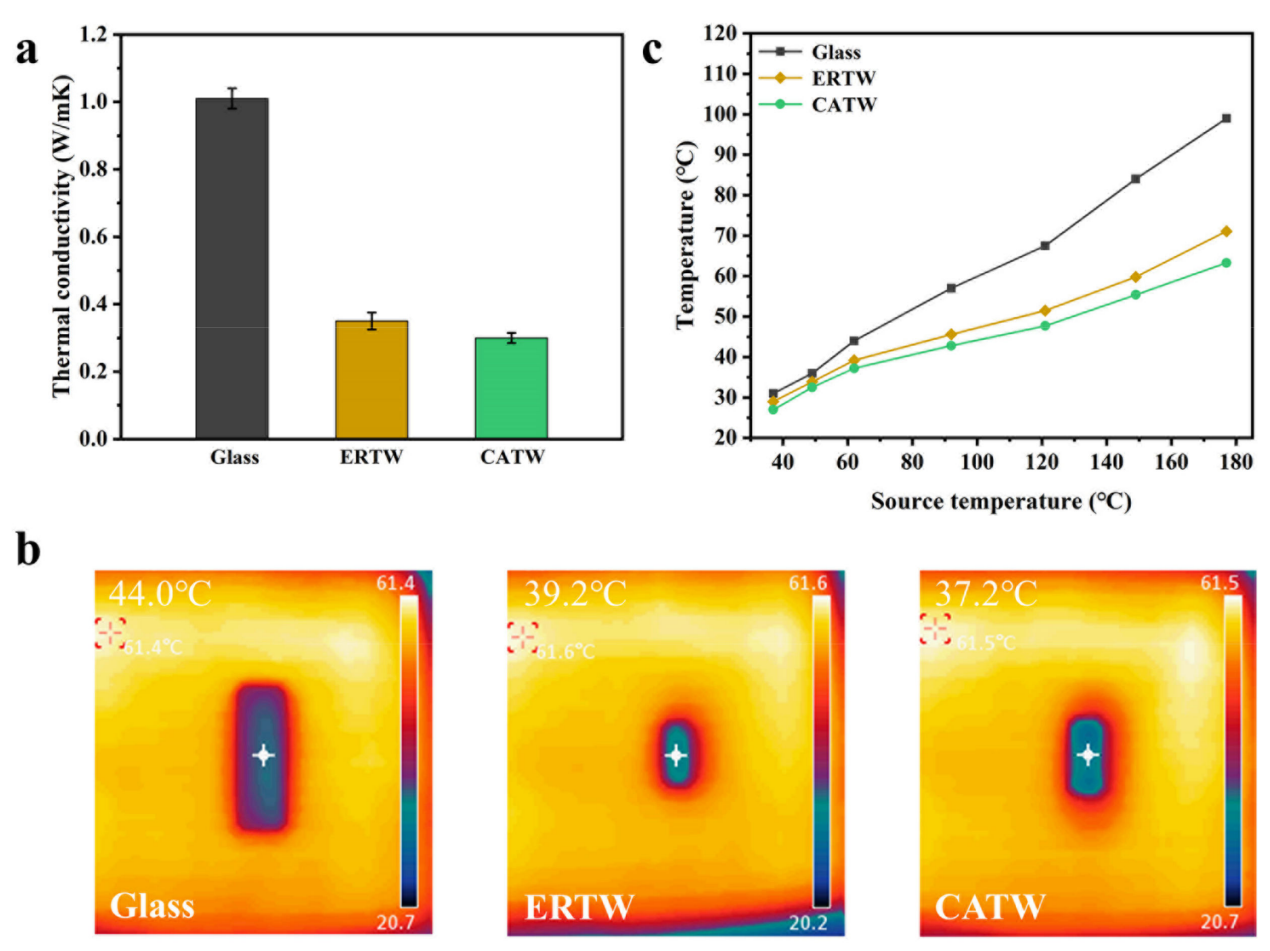
(**a**) Thermal conductivity of glass, epoxy resin transparent wood (ERTW), and CATW. (**b**) Thermal images of glass, ERTW, and CATW. (**c**) Stable backside temperatures at different temperatures through direct contact of glass, ERTW, and CATW with conductive heat sources.

In addition to the optical and mechanical requirements discussed earlier, another important issue in the development of an effective alternative to glass windows is the thermal insulation capability of the material. In this study, transparent wood with a low thermal conductivity was designed to effectively prevent heat dissipation. The thermal conductivity of transparent wood was also investigated. According to the literature, CATW was compared to glass and to the conventional epoxy resin transparent wood (ERTW), as shown in Figure 3a. The thermal conductivity of CATW (~0.30 Wm^−1^K^−1^) was smaller than that of glass (~1.00 Wm^−1^K^−1^) and ERTW (~0.35 Wm^−1^K^−1^) [23]. The lower thermal conductivity of transparent wood may have been due to the high phonon resistance through the wood cell walls (mainly cellulose and hemicellulose) and the multi-interface phonon scattering effect. This finding suggests that it has great potential for window replacement and energy savings.

The three samples mentioned earlier were placed under a conductive heat source and data were collected with an infrared thermometer to further test the thermal management capabilities of the transparent wood (Figure 3b). The infrared thermal image in Figure 3b shows that placing the lower surface of the sample in direct contact with a conductive heat source of approximately 62.0 °C resulted in a temperature of 44.0 °C on the upper surface of the glass, 39.2 °C on the upper surface of the ERTW, and 37.2 °C on the upper surface of the CATW. This result shows that the transparent wood in this work demonstrated good thermal insulation properties. To better illustrate the thermal insulation effect of this transparent wood, the stable backside temperature of the samples was measured after applying different temperatures from 37 °C to 180 °C using the method described earlier (Figure 3c). These results confirmed that the transparent wood in this work is an excellent insulating material and can be a candidate for use in energy-efficient buildings.

**Figure 4:**
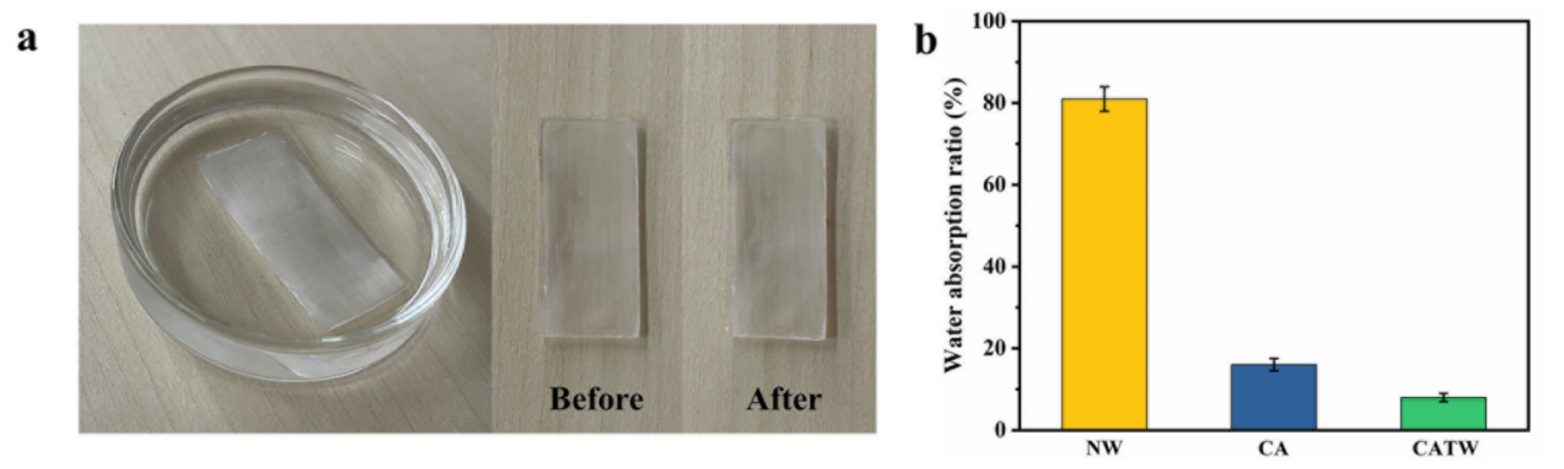
(**a**) Comparison of CATW samples before and after 72 h immersion in water. (**b**) Water absorption ratio of NW, CA, and CATW.

The water resistance of transparent wood was also studied for practical application as a construction material. The water absorption ratios of the NW, CA, and CATW samples were tested by immersing the CATW samples in water, as shown in Figure 4a. The initial masses of the samples (m_1_) weighed were 0.38 g for NW, 2.67 g for CA, and 2.17 g for CATW. After 72 h, the masses of the samples (m_2_) were 0.69 g for NW, 3.11 g for CA, and 2.34 g for CATW. The water absorption ratio was calculated according to Equation (2).

Water absorption ratio (%) = (m_2_ − m_1_)/m_1_ × 100% (2)

where m_1_ is the initial mass (g) of the sample and m_2_ is the mass (g) of the sample after 72 h of water absorption.

Figure 4b shows the water absorption ratio of the samples, which was up to 81.6% for wood, about 16.5% for CA, and about 7.8% for CATW, and Figure 4a shows that CATW did not show any shape distortion or degradation of optical properties. These results demonstrated that the transparent wood in this work had some water repellent properties and that the effect of water on the performance of transparent materials was very small, which shows good application prospects.

## 4. Conclusions

A H_2_O_2_ solution steaming delignification method was developed to remove lignin from wood to obtain delignified wood with a very low lignin content (0.81%). Compared with the previous H_2_O_2_ steaming method, the reaction time and the used H_2_O_2_ volume of the presented H_2_O_2_ solution steaming method were reduced by 37.3% and 52.7%, respectively.

The CATW showed both a high haze (91.0%) and a high light transmittance (83.0%), leading to potential applications in antiglare windows. The CATW had a good tensile strength (56.2 MPa) and modulus (1.87 GPa), suitable for daily applications. The CATW possessed a low thermal conductivity (0.30 Wm^−1^K^−1^), which was lower than those of glass (1.00 Wm^−1^K^−1^) and transparent wood filled with epoxy resin (0.35 Wm^−1^K^−1^).

## Data Availability

The data presented in this study are available on request from the corresponding author.
